# Supplementary material for: Collaborative intelligence in AI: Evaluating the performance of a council of AIs on the USMLE
Source: PLOS Digit Health. 2025 Oct 9;4(10):e0000787. doi: 10.1371/journal.pdig.0000787 (PMC12510544; doi:10.1371/journal.pdig.0000787)
Supplement: S2 File — (ZIP) [file pdig.0000787.s002.zip › S2 Data Raw data files containing unprocessed question inputs and LLM outputs including transcripts of deliberations/STEP3_Question-98-1_Type-MC-NJ.docx]

**Facilitator** **[Round 1]:** A 46-year-old woman comes to the office because of a 4-month history of irregular vaginal bleeding that occurs every 2 weeks and lasts 3 to 5 days. Until 5 months ago when she missed a period, her periods had occurred at regular 30-day intervals and lasted for 5 days. She has not had vaginal pain or discharge, headache, dizziness, chest pain, or shortness of breath. Medical history is unremarkable. She takes no medications. Vital signs are temperature 37.0°C (98.6°F), pulse 80/min, respirations 16/min, and blood pressure 120/60 mm Hg. Pelvic examination discloses normal external female genitalia. There is a small amount of blood in the cervical os. Uterus is 10 cm, mobile, anteverted, nontender, and irregular in shape. Adnexa are without masses or tenderness. Hemoglobin concentration is 10.2 g/dL. Which of the following is the most appropriate next step in evaluation? (A) Determination of serum thyroid-stimulating hormone and prolactin concentrations (B) CT scan of the pelvis (C) Endometrial biopsy (D) Hysteroscopy with dilatation and curettage (E) Trial of hormone replacement therapy

**AI_B:** RESTATING THE QUESTION: This 46-year-old woman has been experiencing about four months of biweekly, irregular vaginal bleeding. While she previously had regular 30-day menstrual cycles, now her bleeding lasts 3 to 5 days. Despite normal vitals and no other notable symptoms, a pelvic examination found some blood in the cervical os and an irregularly shaped, though otherwise normal, uterus. A low hemoglobin concentration could also indicate anemia. We're asked to suggest the most appropriate action to take next based on the provided options. PLANNING: Presuming that this abnormal bleeding is driven by physiological or anatomical issues, we are considering investigating hormone levels, employing imaging to inspect the pelvis, biopsying the lining of the uterus, performing a hysteroscopy with dilatation and curettage (a type of uterine exploration and possible treatment), or starting hormone replacement therapy. CLINICAL REASONING: Irregular vaginal bleeding in a woman in this age group may be due to many causes, including hormonal imbalances, fibroids, adenomyosis, structural abnormalities, or, importantly, precancerous or cancerous conditions. Given the patient’s age and irregular uterine contour, the immediate concern lies with ruling out endometrial carcinoma, which is associated with postmenopausal bleeding or irregular bleeding in women nearing menopause. Normal thyroid-stimulating hormone or prolactin levels will not rule out endometrial cancer. A CT scan of the pelvis won't necessarily provide the detail needed to view the inner lining of the uterus. Hormone replacement offers a therapeutic approach but doesn't allow for diagnostic investigations. CONCLUDING: Considering the need for ruling out endometrial cancer, a definitive diagnosis can be obtained by directly examining the cells of the uterus. Therefore, starting a diagnostic procedure where abnormal tissues can be biopsied for histological examination would be prudent. RESPONSE: The most appropriate next step in evaluation would be (C) an endometrial biopsy. This would allow direct examination of the cells, which is crucial for the diagnosis and exclusion of endometrial carcinoma.

**AI_C:** (1) RESTATING THE QUESTION: A 46-year-old woman, who until 5 months ago had regular menstrual cycles, is experiencing irregular and prolonged bleeding episodes. Despite her reassuring vital signs and an unremarkable medical history, she does have a small amount of blood in her cervical os as well as an irregular uterus, as determined through a pelvic examination. Her anemia further justifies our concern. She lacks systemic symptoms such as headaches, dizziness, or chest pain and she has not reported any kind of pain or discharge in the vaginal area. The query in focus is about what should be the next course of action in this patient's management. (2) PLANNING: The woman's sudden irregularity in menstrual cycles and her age may hint towards the onset of perimenopause or a potentially pathogenic condition such as endometrial hyperplasia or carcinoma. Thus, it is clinically prudent to further evaluate her condition based on these assumptions. (3) CLINICAL REASONING: The patient's gynecological examination revealed an irregular-shaped uterus and presence of blood in her cervical os. Additionally, the woman's hemoglobin concentration was slightly reduced, indicating possible chronic blood loss. The absence of a palpable mass in her adnexa rules out the possibility of large fibroids or ovarian mass. The regularity of her menstrual cycles prior to the last few months and her current age initiates the suspicion of endometrial pathology, possibly due to hormonal changes such as perimenopause or even an early stage of endometrial neoplasia. (4) CONCLUDING: Given this background, the next most appropriate step in the diagnosis is an investigation that could potentially diagnose any endometrial abnormality. Out of the given options, an endometrial biopsy is the test of choice. It can detect hyperplasia or carcinoma, could yield results faster, and is less invasive, avoiding undue surgery at this early stage of the evaluation. (5) RESPONSE: Therefore, the most clinically appropriate next step to evaluate this woman with recent onset irregular menstrual bleeding and slight anemia would be (C) an endometrial biopsy.

**AI_D:** 1) RESTATING THE QUESTION: We're asked to identify the most appropriate next step in evaluating a 46-year-old woman with a recent history of irregular vaginal bleeding that's been occurring every 2 weeks and lasting for 3 to 5 days. The woman's past periods were consistently every 30 days until 5 months ago when she missed a period. No symptoms of systemic illness are present; the pelvic exam revealed a uterus that is mobile, anteverted, non-tender, and irregular in shape and mild anemia. 2) PLANNING: Identifying the chief complaint, i.e., irregular vaginal bleeding with a changed pattern from the woman's regular menstrual cycle coupled with an irregular uterine contour and mild anemia, this suggests a differential diagnosis that includes endometrial pathology like polyps, submucosal fibroids, endometrial hyperplasia, and in the worst case, endometrial cancer. We are tasked with choosing the next best investigative approach. We could measure hormone levels, conduct imaging of the pelvis, obtain a sample of the endometrium for biopsy, perform hysteroscopy with dilatation and curettage, or initiate hormonal therapy. 3) CLINICAL REASONING: Age plays a significant role in the risk of endometrial pathology. Frequent and irregular bleeding that begins in a woman who is perimenopausal or postmenopausal often suggests gynecologic malignancy until proven otherwise. As this patient is 46, she falls into the perimenopausal age group, and the absence of significant pelvic pain or discharge rules out infectious causes. Moreover, the physical examination findings of an irregular uterine contour make benign causes like polyps or fibroids less likely. The primary concern here is endometrial cancer. A CT scan, hormonal testing, and hormone replacement therapy would not be definitive or first-line for diagnosing this concern, making these options less appropriate. 4) CONCLUDING: Therefore, in a woman of this age and with irregular uterine contour on examination and irregular vaginal bleeding, endometrial pathology, including malignancy, is a possible assumption. The best way to evaluate this would be to obtain a sample directly for histological examination. 5) RESPONSE: Hence, the most appropriate next step in evaluation is choice (C), an Endometrial biopsy.

**AI_E:** (1) RESTATING THE QUESTION: The query at hand is about a 46-year-old woman experiencing a 4-month history of abnormal vaginal bleeding that happens around every two weeks and lasts for approximately 3 to 5 days. Her menstrual history prior to this change was regular, with a cyclical pattern occurring every 30 days. Her other medical history is insignificant, and she currently takes no medications. Clinically, she remains free of symptoms like chest pain or vaginal pain; vital signs and pelvic examination reveal normal findings with the exception of irregular uterine shape and minor bleeding in the cervical os. Given these circumstances, what is the best subsequent course of action for a definitive diagnosis? (2) PLANNING: One of the assumptions to consider is that her irregular bleeding could be due to a systemic hormonal imbalance. This could suggest the need to investigate her serum thyroid-stimulating hormone and prolactin concentrations (Option A). Another assumption could be that an unidentified pelvic growth or lesion is causing the irregular bleeding, which might be detected by a CT scan (Option B). Alternatively, the cause of the bleeding may be confined to the endometrial lining itself, perhaps a hyperplasia or malignancy, which could be detected by endometrial biopsy (Option C), or by Hysteroscopy with dilatation and curettage (Option D). Finally, it can also be postulated that this irregular bleeding might reflect perimenopause or ovarian aging, which could potentially be managed with hormone replacement therapy (Option E). It is essential to clinically prioritize the assumption that the irregular bleeding is due to changes in the endometrium because of the woman's age and the physical examination findings of an irregular uterus. (3) CLINICAL REASONING: The nature and frequency of the bleeding, combined with the patient's age, raise suspicion of a possible endometrial pathology. This woman is in the age group where certain conditions, such as endometrial hyperplasia or carcinoma, become more prevalent. The normalization of her menstrual cycle prior to these irregularities further supports this theory. While systemic hormonal imbalances and unidentified pelvic growths could be possibilities, they do not align well with the given findings. Additionally, initiating hormone replacement therapy without a clear diagnosis could be premature and potentially detrimental if an endometrial pathology exists. (4) CONCLUDING: To rule out conditions like endometrial hyperplasia or carcinoma given her age and symptoms, the most appropriate next step is to perform an investigation directly targeting the endometrium. (5) RESPONSE: Therefore, the most suitable course of action under these circumstances would be the endometrial biopsy (Option C) for a direct and effective approach toward determining the underlying cause of this woman's irregular vaginal bleeding.

**AI_A:** (1) RESTATING THE QUESTION: The query presents a case of a 46-year-old female who has been experiencing irregular vaginal bleeding for the past 4 months. Her periods, which used to be regular 5 months ago, now occur every two weeks and last 3 to 5 days. She presents no other vaginal or systemic symptoms, and her medical background is otherwise non-remarkable. Her vital signs and hematological parameters are within normal limits except for the slightly low hemoglobin concentration. During her pelvic examination, her external genitalia appear normal, but an irregularly shaped, 10 cm, mobile, anteverted uterus with a small amount of blood in the cervical os was noted. This means her uterus is tilted forward and there's blood at the opening of her cervix. Adnexa was observed to be free of masses or tenderness. In light of this presentation, the question is asking for the best next step in evaluating her condition. (2) PLANNING: The clinical presentation indicates possible uterine pathology, most probably of endometrial origin, given the irregular bleeding and irregular shape of the uterus. While non-gynecological causes like thyroid abnormalities may cause bleeding irregularities, the patient does not present other signs of hormonal imbalance, making this less likely. Similarly, given the absence of other abdominal or pelvic symptoms or signs, a CT scan of the pelvis is also a less compelling choice. A trial of hormone replacement therapy, while potentially useful for managing irregular bleeding, does not directly evaluate the cause of the problem. (3) CLINICAL REASONING: The irregularity in the vaginal bleeding and the absence of other systemic symptoms make conditions such as endometrial hyperplasia or malignancy a strong consideration. This would also explain the irregular shaped uterus found on the pelvic exam. The next logical reasoning step would be to confirm this suspicion by obtaining a tissue sample of the endometrium for microscopic evaluation. (4) CONCLUDING: Based on a step-by-step explanation of the clinical picture, the most appropriate choice for further evaluation would be to perform an endometrial biopsy. This procedure would provide a tissue sample that would allow for histological evaluation, thus providing potential insight into the exact nature of the pathology causing the irregular vaginal bleeding. Thus, leading to a more focused treatment intervention. (5) RESPONSE: Accordingly, the most appropriate next step in evaluation is C: Endometrial biopsy.

**Facilitator** The consensus among the AI responses is that the most appropriate next step in evaluating the 46-year-old woman's condition is to perform an endometrial biopsy (Option C). This conclusion is based on her age, the irregularity of her menstrual cycle, and the physical examination findings of an irregularly shaped uterus. These factors raise the suspicion of possible endometrial pathology, such as endometrial hyperplasia or carcinoma. While other options like hormonal testing, CT scan of the pelvis, hysteroscopy with dilatation and curettage, or hormone replacement therapy could be considered, they are deemed less appropriate given the current clinical picture. An endometrial biopsy would allow for a direct examination of the cells of the uterus, providing a more definitive diagnosis and potentially ruling out endometrial carcinoma. Therefore, the consensus is that an endometrial biopsy is the most suitable course of action under these circumstances.
